# Functional Transient Receptor Potential Ankyrin 1 and Vanilloid 1 Ion Channels Are Overexpressed in Human Oral Squamous Cell Carcinoma

**DOI:** 10.3390/ijms23031921

**Published:** 2022-02-08

**Authors:** Fruzsina Kiss, Viktória Kormos, Éva Szőke, Angéla Kecskés, Norbert Tóth, Anita Steib, Árpád Szállási, Bálint Scheich, Balázs Gaszner, József Kun, Gábor Fülöp, Krisztina Pohóczky, Zsuzsanna Helyes

**Affiliations:** 1 Somogy County Kaposi Mór Teaching Hospital Kaposvár, H-7400 Kaposvár, Hungary; k.fruzsina17@gmail.com (F.K.); fulopgabor@kmmk.hu (G.F.); 2 Department of Pharmacology and Pharmacotherapy, University of Pécs Medical School, H-7624 Pécs, Hungary; viktoria.kormos@aok.pte.hu (V.K.); eva.szoke@aok.pte.hu (É.S.); angela.kecskes@aok.pte.hu (A.K.); toth.norbert@pte.hu (N.T.); steib.anita88@gmail.com (A.S.); jkun80@gmail.com (J.K.); pohoczkykriszti@gmail.com (K.P.); 3 Centre for Neuroscience, University of Pécs János Szentágothai Research Centre, H-7624 Pécs, Hungary; 4 1st Department of Pathology and Experimental Cancer Research, Semmelweis University, H-1085 Budapest, Hungary; szallasiarpad@gmail.com (Á.S.); scheich.balint@gmail.com (B.S.); 5 Research Group for Mood Disorders, Department of Anatomy & Centre for Neuroscience & Szentágothai Research Centre, University of Pécs Medical School, H-7624 Pécs, Hungary; balazs.b.gaszner@aok.pte.hu; 6 Bioinformatics Research Group, University of Pécs János Szentágothai Research Centre, H-7624 Pécs, Hungary; 7 Department of Pharmacology, Faculty of Pharmacy, University of Pécs, H-7624 Pécs, Hungary; 8 PharmInVivo Ltd., H-7629 Pécs, Hungary

**Keywords:** RNAscope, radioactive _45_Ca^2+^ uptake, cell viability, ATP-based luminescence, oral squamous cell carcinoma, diagnostic and prognostic biomarker, AITC, capsaicin, TRPA1, TRPV1

## Abstract

Oral squamous cell carcinoma (OSCC) is a common cancer with poor prognosis. Transient Receptor Potential Ankyrin 1 (TRPA1) and Vanilloid 1 (TRPV1) receptors are non-selective cation channels expressed on primary sensory neurons and epithelial and immune cells. TRPV1 mRNA and immunopositivity, as well as TRPA1-like immunoreactivity upregulation, were demonstrated in OSCC, but selectivity problems with the antibodies still raise questions and their functional relevance is unclear. Therefore, here, we investigated TRPA1 and TRPV1 expressions in OSCC and analyzed their functions. *TRPA1* and *TRPV1* mRNA were determined by RNAscope in situ hybridization and qPCR. Radioactive _45_Ca^2+^ uptake and ATP-based luminescence indicating cell viability were measured in PE/CA-PJ41 cells in response to the TRPA1 agonist allyl-isothiocyanate (AITC) and TRPV1 agonist capsaicin to determine receptor function. Both *TRPA1* and *TRPV1* mRNA are expressed in the squamous epithelium of the human oral mucosa and in PE/CA-PJ41 cells, and their expressions are significantly upregulated in OSCC compared to healthy mucosa. TRPA1 and TRPV1 activation (100 µM AITC, 100 nM capsaicin) induced _45_Ca^2+^-influx into PE/CA-PJ41 cells. Both AITC (10 nM–5 µM) and capsaicin (100 nM–45 µM) reduced cell viability, reaching significant decrease at 100 nM AITC and 45 µM capsaicin. We provide the first evidence for the presence of non-neuronal TRPA1 receptor in the OSCC and confirm the expression of TRPV1 channel. These channels are functionally active and might regulate cancer cell viability.

## 1. Introduction

Head and neck squamous cell cancers (HNSCC) are the seventh most common cancer types worldwide and almost half of them are oral squamous cell carcinoma (OSCC). They show high mortality, poor prognosis and are often diagnosed at an advanced stage, because of their anatomical situation [1]. Therefore, in most cases, they are no longer amenable to complete surgical removal. The oral mucosa is exposed to various stimuli, including hot, cold and spicy foods/drinks, cigarette smoking and alcohol [2,3], and the latter two appear to have an interactive, multiplicative effect on the risk to develop OSCC [4,5]. Viral infections, for example human papillomavirus, are also important risk factors of the disease [6].

Among the Transient Receptor Potential (TRP) family, several channels, such as TRP Ankyrin 1 (TRPA1) and Vanilloid 1 (TRPV1), have been suggested to play roles in many cancer types, including breast [7,8,9], digestive [10,11], gliomas [12], lung [13], prostate [14] and HNSCC [15]. The Ca^2+^-permeable non-selective cation channel TRPA1 can be activated by hallmark of exogenous stimuli, such as noxious cold, mechanical stimuli and environmental irritants, such as carvacrol, acrolein, hypertonic solutions and pharmacological agents, electrophilic pungent ingredients of mustard oil and garlic (allyl-isothiocyanate: AITC, allicin), cinnamaldehyde and thymol [16,17,18,19,20,21]. Furthermore, it is also activated by endogenous inflammatory mediators and oxidative agents, such as hydrogen peroxide and methylglyoxal, as well as bacterial endotoxins, for example lipopolysaccharide [16,22]. TRPA1 is expressed in sensory neurons and co-localizes with TRPV1, calcitonin gene-related peptide (CGRP) and tachykinins-like substance P (SP) and neurokinin-A [16,23,24,25]. CGRP release leads to vasodilation, whereas tachykinins increase vascular permeability, plasma extravasation and leukocyte migration and induce inflammation. Therefore, TRPA1 acts as a molecular integrator of various stimuli to mediate sensation, pain and neurogenic inflammation [26]. Similarly, to TRPA1, TRPV1 can be activated by various stimuli, including heat (>43 °C), protons (pH ≤ 5.9), capsaicin, cannabinoids, endogenous lipids, and inflammatory mediator (bradykinin, inflammatory cytokines, etc.) [27,28].

TRPA1 expression was shown in cultured human airway epithelial and smooth muscle cells, as well as gingival fibroblasts [22,29]. TRPA1 activation, sensitization and SP release induced and maintained colitis in mice [30,31]. TRPA1 activation by AITC promoted the survival and proliferation of human small cell lung cancer cell line [13]. It was shown that TRPA1 inhibits Panc-1 pancreatic ductal adenocarcinoma cell line cells migration both via channel dependent and independent pathways [32]. TRPA1 upregulation was detected by immunohistochemistry in nasopharyngeal carcinoma patients, which was negatively predictive for disease-specific, distal metastasis- and local recurrence-free survivals [33,34].

Non-neuronal TRPV1 expression was reported in various cutaneous, bladder and airway cell types [35,36,37], with important roles in the growth and differentiation of the human skin [38]. The majority of the TRPV1 expression data are based on immunohistochemical staining [15,39], which, unless supported by functional and/or genetic studies, should be handled with great caution due to the lack of specific antibodies [40]. The same consideration also applies to TRPA1 [33].

Importantly, in human oral epithelial cells capsaicin elevated intracellular Ca^2+^ levels, demonstrating functional TRPV1 expression [41]. TRPV1 expression and immunopositivity were described in all sites of the human oral cavity together with other TRPV channels, and TRPV1 was overexpressed in the tongue and oral floor compared to the gingiva and buccal mucosa [15]. Sparse and weak TRPV1-like immunoreactivity was shown in the basal layer of the healthy human tongue, which was elevated in all layers of the epithelium, both in leukoplakia and tongue cancer. TRPV1 was also expressed on cultured human tongue squamous carcinoma-derived CAL27 cells [39]. TRPV1 upregulation was also detected in esophagitis and esophageal squamous cell carcinoma [42]. 

TRPV1 mRNA and immunopositivity were downregulated in renal cell carcinoma compared to the healthy kidney [43]. TRPV1 protein elevation was described in primary gastric and breast cancer [8,10,11], where it occurs as a potential negative prognostic marker [44]. 

Although several papers are available on TRPV1 expression in the oral mucosa under normal and pathological conditions, there is only one paper about TRPA1 immunopositivity in nasopharyngeal carcinoma [33]. 

Therefore, in the present study, we investigated the TRPA1 and TRPV1 mRNA expressions in the healthy human oral mucosa and OSCC samples using a highly specific and reliable RNAscope in situ hybridization technique. Furthermore, the functional relevance of these ion channels was investigated by radioactive _45_Ca^2+^ uptake and ATP luminescence-based viability assay. 

## 2. Results

### 2.1. TRPA1 and TRPV1 mRNAs Are Present in Healthy Human Oral Mucosa Epithelial Cells, OSCC and PE/CA-PJ41 Cell Line 

**Figure 1:**
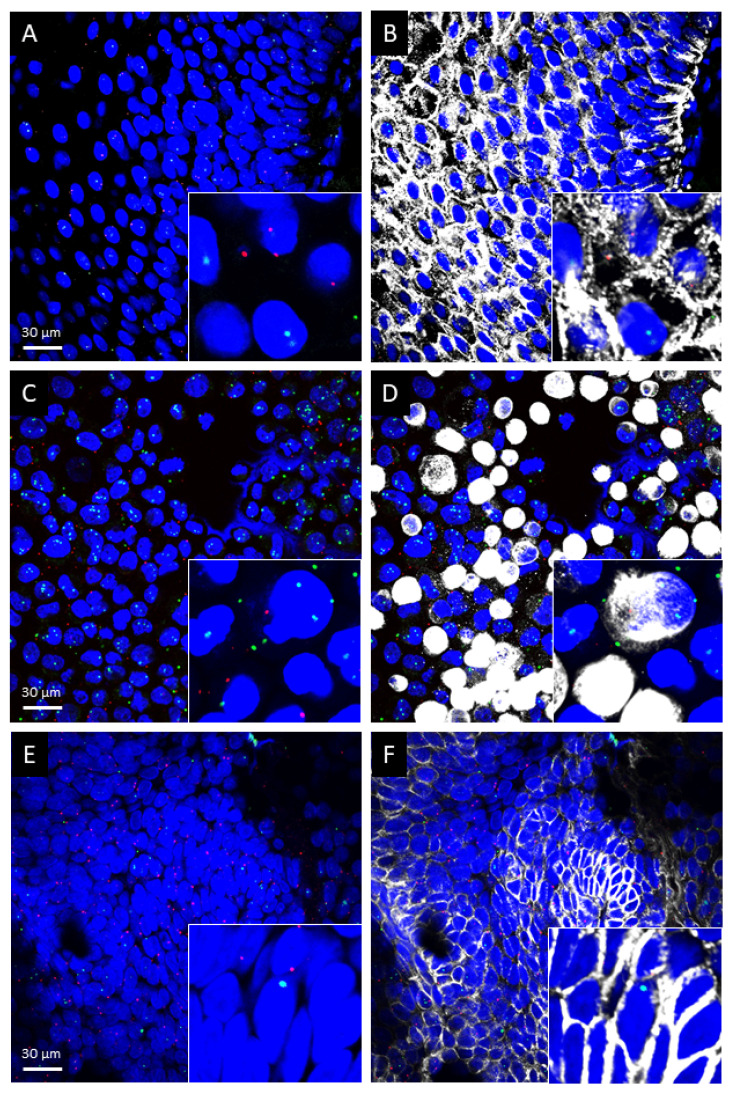
Representative images of *Transient Receptor Potential Ankyrin 1* (*TRPA1*) and *Vanilloid 1* (*TRPV1*) mRNA in normal human oral epithelium (**A**,**B**), in the PE/CA-PJ41 cell line (**C**,**D**) and human squamous cell carcinoma (**E**,**F**). *TRPA1* mRNA (red) and *TRPV1* mRNA (green) by RNAscope and citokeratin-14 protein (white) by immunofluorescence were depicted and counterstained with DAPI (blue) for nuclei. Scale bar: 30 µm for all images.

Confocal laser scanning microscopy and qualitative morphological assessment revealed that both *TRPA1* and *TRPV1* mRNAs are expressed in the squamous epithelium of the healthy oral mucosa (n = 3, Figure 1A,B) and OSCC (n = 6, Figure 1E,F) samples. In agreement with this finding, both mRNAs were detected in PE/CA-PJ41 cells as well. Nearly all *TRPA1-* and *TRPV1*-positive cells contained the epithelium-specific keratinocyte marker citokeratin-14 in all samples (Figure 1C,D).

TRPA1-like immunopositivity was observed in the basal and prickle cell layers of the healthy human oral mucosa, mainly in the cytoplasmic, but also in the nuclear regions. In most cancer samples, particularly in the poorly differentiated cases, TRPA1 immunopositivity was substantially stronger in the epithelial, vascular endothelial and some lymphoid cells, but the staining was mainly located in the nuclei, clearly suggesting a non-specific reaction. The red and green refer to the computer-generated image analysis (ImageQuant, 3DHistech) artificial coloring, which are proportional to the intensity of the immunopositivity in a scale where red represents the strongest and blue no staining (Figure S1). These, of course, do not provide information about the specificity of the antibody. Furthermore, this antibody did not give any signal on CHO cells stably expressing the human TRPA1 receptor (data not shown).

Although several anti-TRPV1 antibodies are commercially available for human tissues (Biorbyt Ltd., Cat. no.: orb251483; Novus Biologicals, Cat. no.: NB100-98886; Abcam plc., Cat. no.: ab3487), none of them have been proven to be specific in our hands, on paraffin-embedded sections. 

### 2.2. TRPA1 and TRPV1 mRNAs Are Significantly Upregulated in OSCC Samples

**Figure 2:**
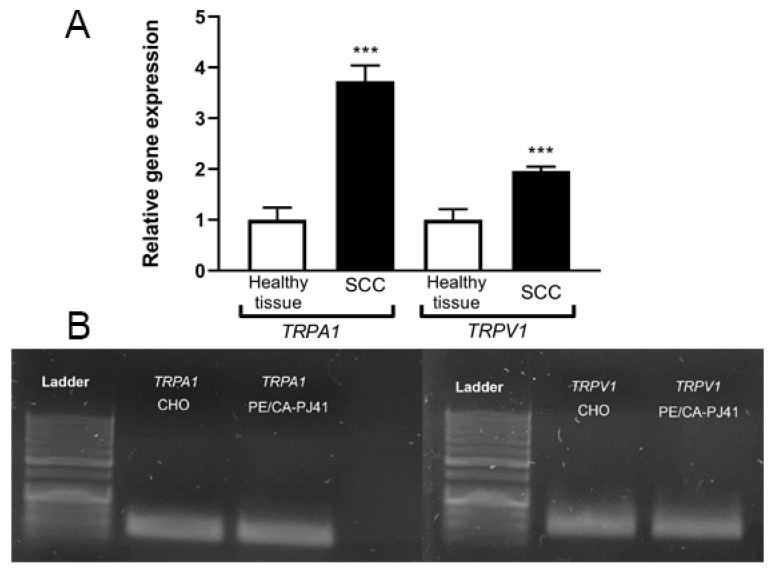
Relative gene expression ratios of *Transient Receptor Potential Ankyrin 1* (*TRPA1*) and *Vanilloid 1* (*TRPV1*) receptors normalized to the *Importin 8 (IPO8)* reference gene in the healthy control oral mucosa (n = 10), compared to oral squamous cell carcinoma (OSCC; n = 15). Columns represent the mean + SEM, *** *p* < 0.001, Mann–Whitney U test; (**A**). Expression of *TRPA1* and *TRPV1* mRNA in the PE/CA-PJ41 cell line and *TRPA1* and *TRPV1* expressing CHO cells (positive controls) (n = 4/group; (**B**)).

In agreement with the RNAscope results, both *TRPA1* and *TRPV1* mRNAs were stably expressed in the healthy human oral mucosa samples (n = 10). Significantly, approximately 4-fold and 2-fold *TRPA1* and *TRPV1* mRNA increases were detectable in OSCC (n = 15) samples, respectively, compared to the healthy control samples (Figure 2A). Both *TRPA1* and *TRPV1* mRNAs were expressed in the OSCC PE/CA-PJ41 cell line (Figure 2B), reaching the threshold cycle of Ct 29.4 and 25.1, respectively, during the qRT-PCR measurement. 

### 2.3. Both TRPA1 and TRPV1 Activations Induce Radioactive _45_Ca^2+^ Uptake in PE/CA-PJ41 Cells 

**Figure 3:**
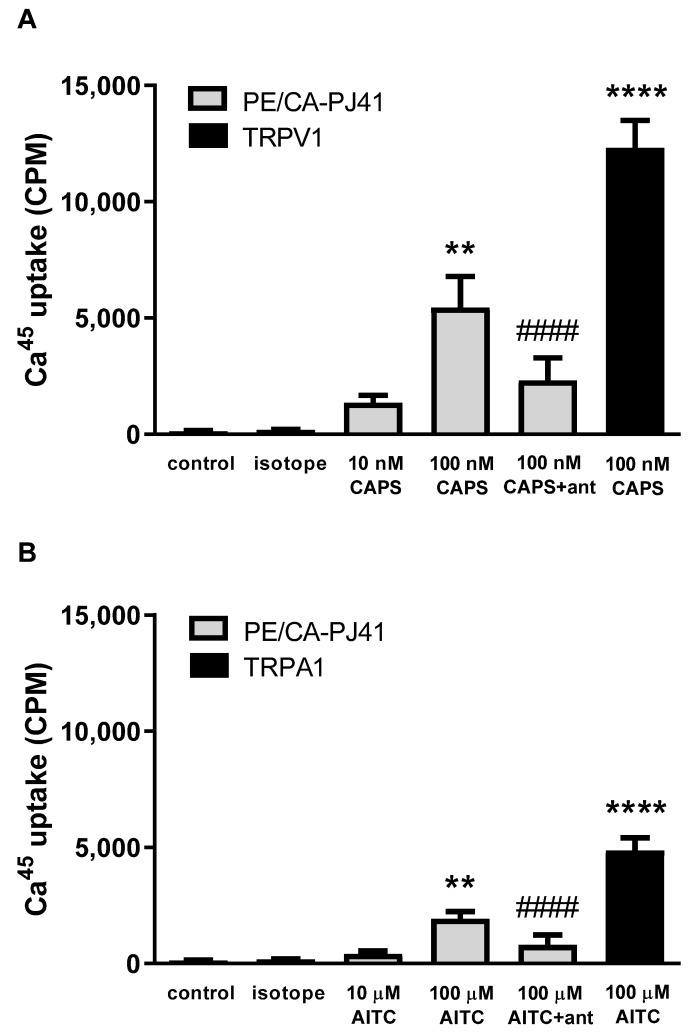
Effect of allyl-isothiocianate (AITC) and capsaicin (CAPS) on _45_Ca^2+^ uptake (count per minute: CPM) of CHO cells expressing the cloned Transient Receptor Potential Ankyrin 1 (TRPA1, (**A**)) and Vanilloid 1 (TRPV1, (**B**)) receptors and PE/CA-PJ41 cells. CAPS and AITC responses were antagonized by capsazepine (10 µM) and HC-030031 (10 µM), respectively. _45_Ca^2+^ accumulations are presented as a percentage of agonist control. Each column represents the mean ± SEM of n = 9, ** *p* < 0.01, **** *p* < 0.0001 (vs. control, one-way ANOVA, Dunnett’s post hoc test); #### *p* < 0.0001 (vs. 100 nM CAPS (**A**) or 100 µM AITC (**B**), one-way ANOVA, Dunnett’s post hoc test).

In order to demonstrate that PE/CA-PJ41 cells express functionally active TRPA1 and TRPV1 ion channels, AITC- and capsaicin-induced _45_Ca^2+^-influx was measured. The TRPA1 agonist AITC (10 and 100 µM) resulted in a concentration-dependent, 418.3 ± 120.2 and 1928 ± 315.8 CPM _45_Ca^2+^-influx into PE/CA-PJ41 cells, respectively. For a positive control comparison, this value was 4874 ± 545.97 on stable TRPA1 receptor-expressing CHO cells in response to 100 µM AITC (Figure 3A). 

The TRPV1 agonist capsaicin (10 and 100 nM) induced a concentration-dependent 1353.3 ± 315.4 and 5443.3 ± 1335.8 CPM _45_Ca^2+^-retention, respectively, in PE/CA-PJ41 cells. This response was 12,324.7 ± 1168.1 CPM on the TRPV1 receptor-expressing CHO cell line for 100 nM capsaicin stimulation (Figure 3B). The retention of _45_Ca^2+^ was negligible; around 100–200 CPM in the Hank’s solution alone and Hank’s solution plus isotope controls (Figure 3). The TRPV1 antagonist capsazepine (CZP, 10 µM) significantly inhibited the capsaicin-evoked, and the TRPA1 antagonist HC-030031 (10 µM) significantly reduced the AITC-induced _45_Ca^2+^-uptake (Figure 3).

### 2.4. Both TRPA1 and TRPV1 Activations Reduce PE/CA-PJ41 Cell Viability 

**Figure 4:**
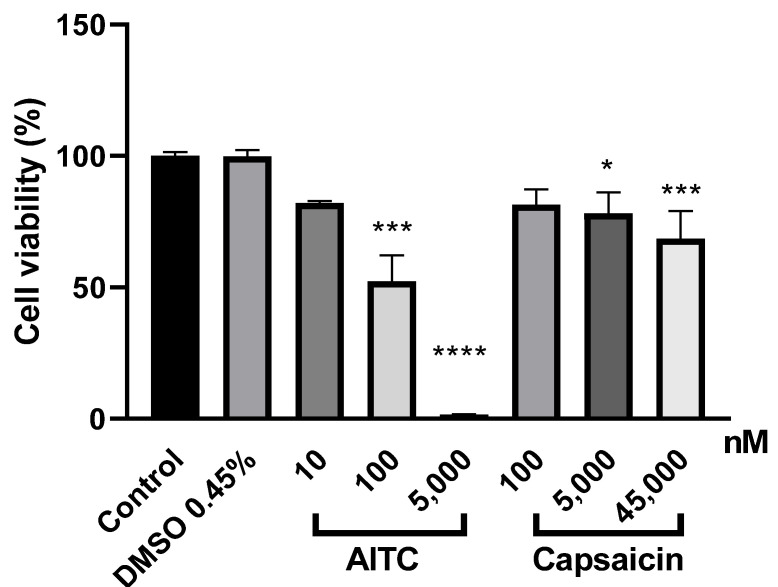
Effect of AITC and capsaicin in comparison with the solvent dimethyl sulfoxide (DMSO) on the viability of PE/CA-PJ41 cells, assessed by the CellTiter-Glo^®^ Luminescent Cell Viability Assay. Data show the average results of three independent experiments ± SEM. * *p* < 0.05, *** *p* < 0.005, **** *p* < 0.0001 (vs. control, one-way ANOVA, Dunnett’s post hoc test).

Incubation PE/CA-PJ41 cells with three different concentrations of the TRPA1 agonist AITC, for 24 h, resulted in a concentration-dependent cell viability decrease with a non-significant 18% reduction in 10 nM, a significant 48% reduction in 100 nM and complete viability loss (99%) in 5000 nM. Meanwhile, capsaicin induced non-significant 19–32% reduction of the cell viability in the concentration range of 100–45,000 nM concentration (Figure 4). In the case of solvent control cells, the vehicle DMSO did not affect the cell viability at its highest concentrations.

## 3. Discussion

We provide the first evidence for the expression and local mRNA upregulation of the *TRPA1* ion channel in OSCC, as well as its functionality in OSCC cell line. Furthermore, our results confirm the expression and upregulation of *TRPV1* mRNA in OSCC. 

*TRPA1* has been described to be upregulated in different tumors, such as nasopharyngeal carcinoma [33], pancreatic adenocarcinoma [32] and prostate cancer-associated fibroblast cell cultures [45]. Elevated TRPV1 expression was shown in breast and prostate cancer [45]. *TRPV1* mRNA and immunopositivity were significantly upregulated in all layers of the epidermis in tongue, bucca, oral floor and gingiva OSCC samples in comparison with the normal oral mucosa [15]. These findings clearly demonstrated *TRPA1* and a lesser extent also *TRPV1* mRNA expressions in keratinocytes, which might have roles in cell growth, survival, inflammatory and abnormal proliferative processes [46,47,48].

The results of TRPA1 and TRPV1 protein expressions demonstrated by immunohistochemistry should be handled with caution, due to the lack of specific antibodies [40]. Our data support this conclusion, since we also found TRPA1 staining non-specific, based on the nucleic signals inconsistent with the highly sensitive and specific. 

We found that both AITC and capsaicin induce Ca^2+^-influx in OSCC cells, demonstrating their functional expression and decreasing their viability. Their activation-induced effect is presumably based on Ca^2+^-mediated mechanisms, which could be addressed in future research. However, our results are in agreement with other findings obtained from HeLa cervical cancer cell line [49,50] and gastric cancer cells [51]. Furthermore, 24 h AITC treatment significantly reduced the viability of OSCC line at 100 nM and completely diminished the viability by 99% at 5 µM concentration. Liu et al. reported that a high dose (≥20 µM) of AITC decreased cell viability, increased DNA damage and inhibited cell migration in HepG2 human hepatocellular carcinoma cells [52]; moreover, AITC has antiangiogenic affect in in vivo studies [53]. A number of studies also showed that TRPA1 agonist AITC negatively affects the cell proliferation of breast [54], bladder [50] and cervix cancer cells [49,50]. 

Data about the role of these receptors in cancer are contradictory: Experimental studies showed that AITC and capsaicin have an inhibitory effect on cell proliferation through apoptosis induction in various cancer cells. In contrast, others reported that both compounds promote tumor formation [45,55]. In human breast cancer cells, AITC induced mitochondrial-mediated apoptosis via cytochrome-c and release of apoptosis inducing factor and endonuclease G, as well as the activation of caspase-9 and caspase-3 [54]. Capsaicin-induced cell toxicity was shown in rat nodose ganglion neurons, by inducing mitochondrial damage and, hence, neuronal damage or death [56]. 

Growing evidence suggests that ion channels including TRP receptors contribute to the development of certain malignancies; thus, these diseases are considered a special type of “channelopathy”, namely, “oncochannelopathy” [57]. TRP channels affect many aspects of cancer progression at all stages by modulating Ca^2+^-regulated cell processes, including proliferation, survival and even tumor formation [55]. In agreement with these, our results suggest that TRPA1 and TRPV1 activations reduce OSCC cell viability, presumably by increasing intracellular Ca^2+^ levels and triggering related signaling pathways. 

The limitation of the present study is that receptor functionality was only determined in the human PE/CA PJ-41 OSCC cell line, since it was technically not possible to perform functional studies on primary cell cultures of OSCC samples. Since this cell line does not mimic all features of OSCC, the conclusion drawn from these experiments should be cautious, but it is still valuable, and the results support the expression data.

Clinically useful TRP modulators might be promising therapeutic agents, but severe systemic side effects of TRPV1 antagonists developed for analgesic purposes, such as hyperthermia, discourage the development of systemically applied candidates. However, topically administered TRPA1 and/or TRPV1 agonists could avoid systemic unwanted effects, and therefore, might provide beneficial treatment potentials on the oral mucosa even against cancer generation and progression.

## 4. Materials and Methods

### 4.1. Study Participants and Tissue Collection

The study was approved by the Regional Research Ethics Committee of the University of Pécs Clinical Centre (license number: 7250-PTE 2018) and Scientific Council for Health Scientific and Research Ethics Committee (ETT-TÜKEB, license number: IV/2983-/2020/EKU).

A total of 15 OSCC patients (9 males, 6 females) were included in the present study, aged between 57 and 92; all of them underwent surgical or experimental resection at the Department of Oral and Maxillofacial Surgery of Somogy County Kaposi Mór Teaching Hospital, Hungary between September 2017 and April 2020 (Table 1). Patients with precancer lesions and adenocarcinoma were excluded. Clinical data, including age, sex and tumor histological type and site, were collected. Oral mucosa samples were harvested under local anesthesia using 1 mL lidocaine-adrenaline injection (2–0.001%). OSCC samples were removed from bucca, lower lip, gingiva, tongue, sublingual region, hard palate and floor of the oral cavity by surgical excision. Diagnosis and certainty of the OSCC was defined by histopathological examination. Control samples of a similar size as the squamous cell carcinoma samples (n = 10) were taken from bucca, lower lip, gingiva and tongue of healthy volunteers by surgical excision.

### 4.2. Human Oral Squamous Cell Carcinoma Cell Line

PE/CA-PJ41 (clone D2) OSCC cell line has been derived from the oral squamous epithelium of a 67-year-old female patient. It was purchased from Merck KGaA (Darmstadt, Germany). It was cultured in a humidified air atmosphere in 5% CO_2_ concentration at 37 °C, in RPMI-1640 w/o l-Glutamine medium (Lonza, Switzerland), supplemented with 10% fetal bovine serum albumin (Merck KGaA, Darmstadt, Germany), 1% non-essential amino acids (Merck KGaA, Darmstadt, Germany), 1% l-Glutamine (Merck KGaA, Darmstadt, Germany) and 0.1% penicillin + streptomycin solution (Lonza, Basel, Switzerland) [58]. 

### 4.3. RNA Isolation and Real-Time Quantitative Polymerase Chain Reaction (RT-qPCR)

In the case of tissue samples, total RNA isolation was performed as follows: samples were placed into 1000 μL TRI-Reagent (Thermo Fischer Scientific, Waltham, MA, USA) and homogenized with stainless steel balls with the help off Tissue Lyser equipment (Qiagen, Hilden, Germany) and cordless pestle motor (VWR International Ltd., Debrecen, Hungary). PE/CA-PJ41 cell line was also homogenized in 1 mL TRI-reagent by vortexing. RNA contents of both sample types were isolated using Direct-zol™ RNA MiniPrep (for tissue lysates) and MicroPrep (for cell lysates, Zymo Research, Irvine, CA, USA), according to the manufacturer’s instructions. The amount and purity of RNA were determined using a Nanodrop ND-1000 Spectrophotometer V3.5 (NanoDrop Technologies, Inc., Wilmington, DE, USA). Samples were treated with 1U DNase I enzyme to eliminate remaining genomic DNA. Tissue cDNA was synthesized from 500 ng RNA with a Maxima First Strand cDNA Synthesis Kit (Thermo Scientific, Waltham, MA, USA), while for cell lysates, an Applied Biosystems™ High-Capacity Reverse Transcription Kit (Thermo Fisher Scientific, Waltham, MA, USA) was used.

Relative gene expression ratios of the human *TRPA1* and *TRPV1* were determined with the QuantStudio™ 5 system (Life Technologies Magyarország Ltd., Budapest, Hungary) in a 96-well block using *importin 8 (IPO8)* as a reference gene [59]. Measurements were performed in triplicates, in a reaction volume of 10 µL, containing 1x SensiFAST™ Probe Lo-ROX mix (Meridiane Bioscience, Memphis, TN, USA), 400 nM probe primer mix (forward and reverse) and 20 ng cDNA. FAM conjugated TaqMan™ Gene Expression Assays (Thermo Scientific, Waltham, MA, USA), which were used to amplify the target loci: *IPO8*: Hs00914057_m1, *TRPA1*: Hs00175798_m1 and *TRPV1*: Hs00218912. Geometric means of the Cq values were calculated of both genes, and the gene expression was calculated using the ΔΔCt method [60].

The PE/CA-PJ41 PCR products were electrophoresed on a 2.5% agarose gel containing 0.01% GelRed (Biotium, Harward, CA, USA) at 70 V for 40 min, and visualized by a Safe View (Cleaver 182 Scientific Ltd., Warwickshire, UK) transilluminator (Figure 2B). 

### 4.4. Tissue Collection and Sample Preparation for RNAscope Study

OSCC samples (n = 6) were harvested from gingiva, tongue and hard palate by surgical excision. Diagnosis and certainty of the OSCC was defined by histopathological examination. Control samples of a similar size as the squamous cell carcinoma samples (n = 3) were taken from gingiva and tongue of healthy volunteers by surgical excision (Table 2). 

Human tissues were post-fixed for 24 h in 10% neutral buffered formalin solution (Merck, Darmstadt, Germany, Cat. No.: HT501128), rinsed in PBS, dehydrated and embedded in paraffin using standard procedures. Sections of 5 µm were cut using a sliding microtome (HM 430 Thermo Fisher Scientific, Waltham, MA, USA).

The pre-treatment of PE/CA cell line was performed according to the RNAscope^®^ Multiplex Fluorescent Reagent Kit v2 (ACD, Hayward, CA, USA) User Manual.

### 4.5. Immunohistochemistry and Image Analysis

For the immunohistochemical examination of the OSCC tissue samples, 5 μm thick sections were deparaffinized and rehydrated. For antigen retrieval, tissue samples were incubated in citrate buffer at pH 6 in a microwave oven for 3 × 5 min at 750 W, and then, were cooled to room temperature (RT). Tissue endogenous peroxidase activity was inhibited by incubation of the sections in 3% hydrogen peroxide (H_2_O_2_) for 20 min. Samples were incubated in normal goat serum for 20 min in order to prevent nonspecific binding of the secondary antibody. Sections were then first incubated at a 1:1000 dilution with rabbit polyclonal anti-TRPA1 antibody (TRPA1: Novus 40763) for 1 h at RT. After several washing steps, sections were incubated with horseradish peroxidase-conjugated anti-rabbit secondary antibodies (EnVision Rabbit HRP Kit, Dako-Cytomation, Carpinteria, CA, USA) for 30 min. Sections from normal mucosa incubated without primary served as negative controls. The TRPA1-like immunopositivity of the OSCC sections was examined under bright-field microscope (Olympus, BX51). Furthermore, we tested this antibody on the stable human TRPA1 expressing CHO cell line (dilution: 1:1000) with Cy5-conjugated anti-rabbit secondary antibody (GYÁRTÓ). The immunopositivity was examined with a Nikon Eclipse XXX fluorescent microscope.

### 4.6. RNAscope In Situ Hybridization Combined with Immunolabeling

The RNAscope Multiplex Fluorescent Reagent Kit v2 (ACD, Hayward, CA, USA) was used according to the manufacturer’s protocol. In short, human sections were heat-treated, deparaffinized, H_2_O_2_-blocked, boiled and pre-treated with Protease Plus. In case of PE/CA-PJ41 cell line, Protease III treatment was performed. Samples were subsequently hybridized with probes specific to human *TRPA1* (ACD, Cat. No. 837411-C2), *TRPV1* (ACD, Cat. No. 415381) mRNA and with 3-plex positive (ACD Cat. No. 320861) and negative (ACD, Cat. No. 320871) control probes. Sequential signal amplification and channel development was performed. Slides were subjected to an immunofluorescent labeling using monoclonal rabbit anti-cytokeratin-14 antiserum (Abcam, EPR17350, Cat. No. ab181595, diluted to 1:500) for 24 h at 24 °C. After 2 × 15 min washes in PBS, Alexa 647-conjugated donkey anti-rabbit (AB_2492288, Jackson Immunoresearch Europe Ltd., Cambridgeshire, UK; Cat. No. 711-605-152, diluted to 1:500,) serum was used for 3 h. Nuclear counterstaining with 4′,6-diamidino-2-phenylindole (DAPI) was performed and sections were mounted with ProLong Diamond Antifade Mountant for confocal imaging. Human 3-plex positive (ACD; Cat. No.: 320881) control probes specific to *POLR2A* mRNA (fluorescein), *PPIB* mRNA (Cy3) and *UBC* mRNA (cyanine 5, Cy5) and 3-plex negative (ACD; Cat. No.: 320871) control probes specific to bacterial *dabP* mRNA were tested in the human squamous cell carcinoma (Figure S2) and PE/CA cell line (Figure S3).

Fluorescent images were acquired using an Olympus Fluoview FV-1000 laser scanning confocal microscope (Olympus, Tokyo, Japan) and the Fluo-View FV-1000S-IX81 image acquisition software system. The confocal aperture was set to 80 µm. The analogue sequential scanning was performed using a 40× objective lens (NA: 0.75). The optical thickness was set to 1 μm and the resolution was 1024 × 1024 pixel. The excitation time was set to 4 µs per pixel. Blue, green, red and white virtual colors were selected to depict fluorescent signals of DAPI (nuclear counterstain), Fluorescein (*TRPV1* mRNA), Cyanine 3 (*TRPA1* mRNA) and Alexa 647 (citokeratin-14 protein), respectively. DAPI was excited at 405 nm, Fluorescein at 488 nm, Cy3 at 550 nm and Alexa 647 at 650 nm.

### 4.7. Radioactive Calcium-45 Uptake Experiments on PE/CA-PJ41 Cells and TRPV1 or TRPA1 Receptor-Expressing CHO Cell Lines 

For receptor selectivity experiments, PE/CA-PJ41 cells and CHO cells stably expressing TRPV1 or TRPA1 ion channels were investigated in response to capsaicin as well as AITC. CAPS and AITC responses were antagonized by capsazepine (10 µM) and HC-030031 (10 µM), respectively. Cells were plated in 15 μL cell culture medium onto Microwell Minitrays (Merck KGaA, Darmstadt, Germany) and incubated overnight at 37 °C in a humid atmosphere with 5% CO_2_. The following day, cells were washed with calcium free Hank’s solution (pH 7.4), and then, they were incubated in 10 μL of the same buffer containing the desired amount of capsaicin (10 nM, 100 nM) or AITC (10 µM, 100 µM) and 200 μCi/mL _45_Ca^2+^ isotope (1.3 Ci/mmole, Ammersham) for 3 min at room temperature. After washing with ECS, the residual buffer was evaporated, the retained isotope was collected in 15 μL 0.1% SDS and the radioactivity was measured in 2 mL scintillation liquid in a Packard Tri-Carb 2800 TR scintillation counter. _45_Ca^2+^ isotope-retention was presented in count per minute (CPM).

### 4.8. Cell Viability Assay

Cell were treated with capsaicin (Thermo Fisher Scientific) and AITC (Merck KGaA, Darmstadt, Germany) dissolved in dimethyl sulfoxide (DMSO) using the following concentrations.

Capsaicin concentration ranged between 100 nM and 45 µM (100 nM, 1 µM, 2.5 µM, 5 µM, 10 µM, 15 µM, 25 µM and 45 µM) and AITC treatments were carried out at 100 nM, 1 µM, 5 µM, 7 µM, 10 µM, 15 µM and 45 µM (n = 6–6) [51]. DMSO concentration of the solvent treated control cells were adjusted to similar concentrations as the highest concentration of the treatments with capsaicin (0.45%, 0.25% and 0.15%) and AITC (0.45%, 0.15% and 0.1%) [49]. 

Cells were seeded in 96-well tissue culture plates and cultured for 24 h. After the addition of AITC and capsaicin, cells were incubated for 24 h and cell viability was assessed using a CellTiter-Glo^®^ Luminescent Cell Viability Assay (CTG, Promega, Madison, WI, USA) mixture, as recommended by the manufacturer. ATP-based luminometric measurement from the metabolically active cells in the culture were determined by EnSpire^®^ Multimode Plate Reader. Viability was calculated using the background-corrected luminescence as follows: Viability (%) = A of experiment well/A of control well × 100.

### 4.9. Statistical Analysis

Statistical analyses were performed using GraphPad Prism 8 software. The distribution of the data was examined with a Kolmogorov–Smirnov normality test, followed by one-way ANOVA and Dunnett’s post hoc test (in the case of normal distribution) or Mann–Whitney U-test (if the data were not normally distributed). In all cases, *p* < 0.05 was considered as statistically significant.

## Figures and Tables

**Table 1:** Summary of the occurrence and demographics of the patients with histologically validated squamous cell carcinoma and control samples (M = male, F = female, HT = hypertonia, RA = rheumatoid arthritis, CLL = chronic lymphoid leukemia, OP = osteoporosis, DM = diabetes mellitus).

Type	Localization	Sex	Age	Comorbidities	Smoking	Alcohol
OSCC	tongue, floor of oral cavity	F	69	HT	yes	no
OSCC	floor of oral cavity	M	65	No	yes	yes
OSCC	bucca	F	57	RA, OP	no	no
OSCC	floor of oral cavity	M	92	DM	no	no
OSCC	tongue	M	67	CLL, HT	yes	yes
OSCC	tongue	M	67	pancreatitis	yes	yes
OSCC	floor of oral cavity	F	56	no	yes	yes
OSCC	tongue	M	53	epilepsy	yes	yes
OSCC	lower lip	M	58	HT, paraplegia	no	no
OSCC	floor of oral cavity	F	68	no	yes	no
OSCC	gingiva	F	63	no	yes	no
OSCC	sublingual region, tongue	M	63	no	yes	yes
OSCC	tongue	F	82	HT	no	no
OSCC	hard palate	M	57	HT	yes	no
OSCC	bucca	M	54	no	yes	yes
normal tissue	bucca	M	64			
normal tissue	bucca	F	33			
normal tissue	lower lip	F	45			
normal tissue	gingiva	F	59			
normal tissue	bucca	F	70			
normal tissue	gingiva	M	65			
normal tissue	lower lip	F	47			
normal tissue	lower lip	M	52			
normal tissue	tongue	F	53			
normal tissue	tongue	F	69			

**Table 2:** Summary of the occurrence and demographics of the patients with histologically validated squamous cell carcinoma and control samples for RNAscope in situ hybridization (M = male, F = female).

Type	Localization	Sex	Age
OSCC	tongue	M	80
OSCC	gingiva	M	53
OSCC	gingiva	M	56
OSCC	tongue	M	68
OSCC	hard palate	M	57
OSCC	tongue	F	72
normal tissue	tongue	M	66
normal tissue	gingiva	F	22
normal tissue	gingiva	M	29

## Data Availability

The data presented in this study are available upon request from the corresponding author.

## Supplementary Materials

The following are available online at www.mdpi.com/article/10.3390/ijms23031921/s1.
