# Supplementary material for: Functional Transient Receptor Potential Ankyrin 1 and Vanilloid 1 Ion Channels Are Overexpressed in Human Oral Squamous Cell Carcinoma
Source: Int J Mol Sci. 2022 Feb 8;23(3):1921. doi: 10.3390/ijms23031921 (PMC8836603; doi:10.3390/ijms23031921)
Supplement: Supplementary file 1 [file ijms-23-01921-s001.zip › ijms-1533783-supplementary.pdf]

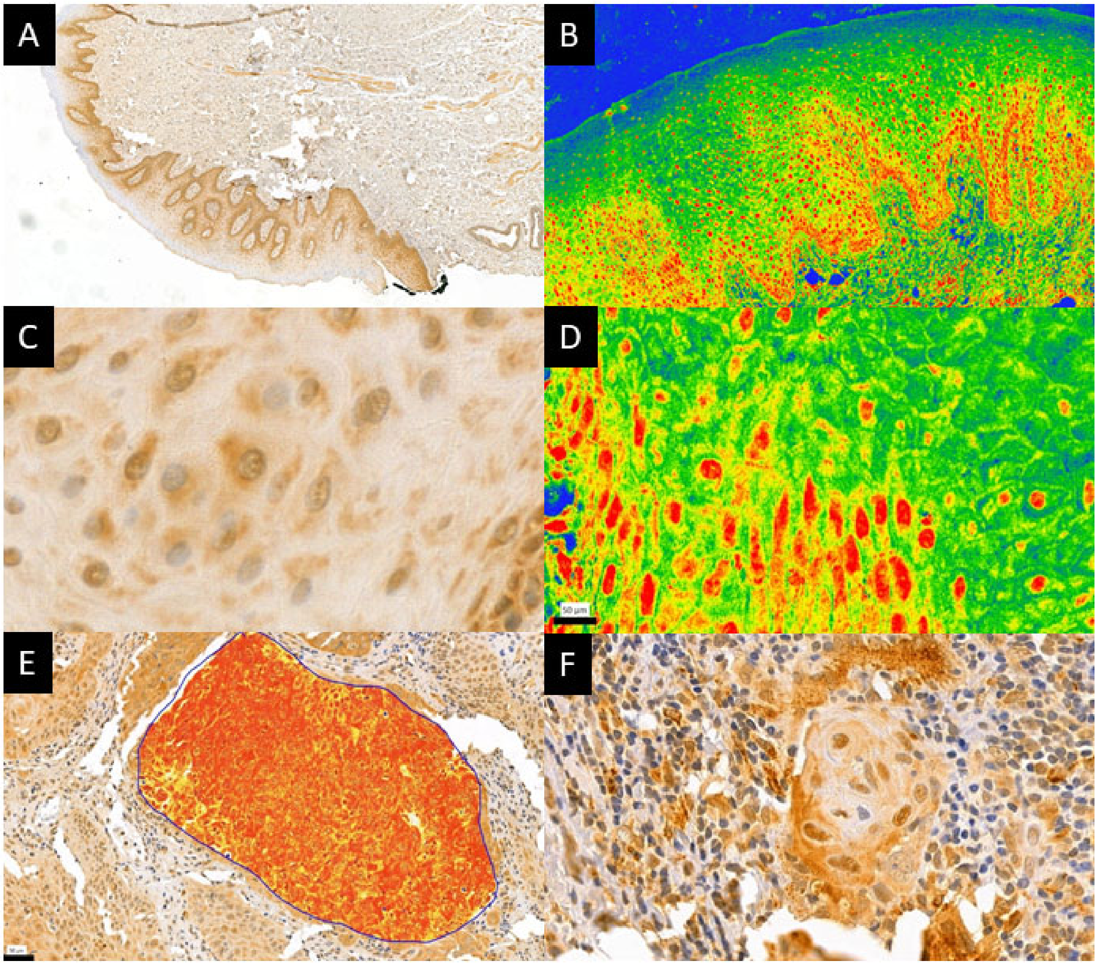

**Figure S1.** Immunostaining with the Novus 40763 anti-TRPA1 antibody in control oral squamous mucosa (**A–D**) and poorly differentiated, invasive oropharyngeal squamous cell carcinoma (**E, F**). In the control tissue, immunostaining in restricted to the basal layer of the epidermis (A, 2X) where the dividing, mitotically active cells are located; in these cells, the immunostaining is both nuclear membrane and cytoplasmic (C, 40X). Immunoreactivity is also present in the vascular endothelium (A). The intensity of the immunoreactivity in panels A and C was analyzed by the ImageQuant program of 3DHistech, Budapest, Hungary. Red indicates strong, and blue means no staining. Panel F shows an invasive nest of squamous cell carcinoma (40X). This nest was identified for the ImageQuant program (E). Note strong and uniform TRPA1-like immunoreactivity in the tumor cells (E and F).

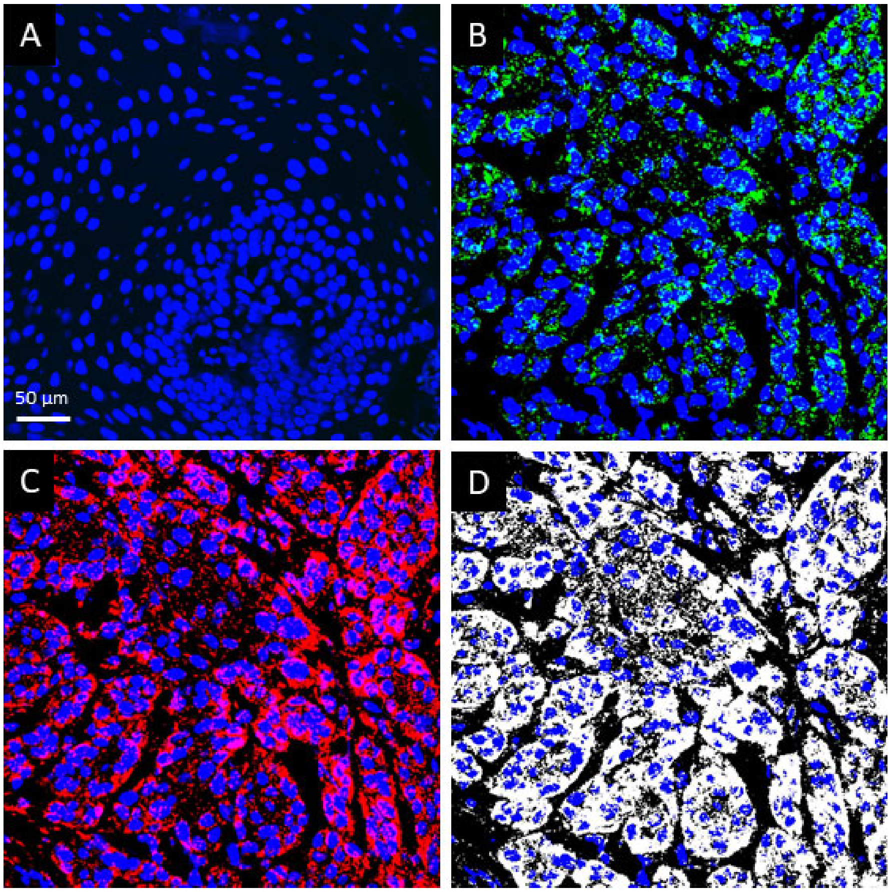


**Figure S2.** RNAscope triplex positive and negative controls in the human squamous cell carcinoma. The RNAscope triplex negative control probes would hybridize with the bacterial dabP gene (**A**). RNAscope triplex positive control probes specific to human *POLR2A* (**B**), *PPIB* (**C**) and *UBC* (**D**) mRNA targets are shown in green, red and white, respectively. Scale bar: 50 µm for all images.

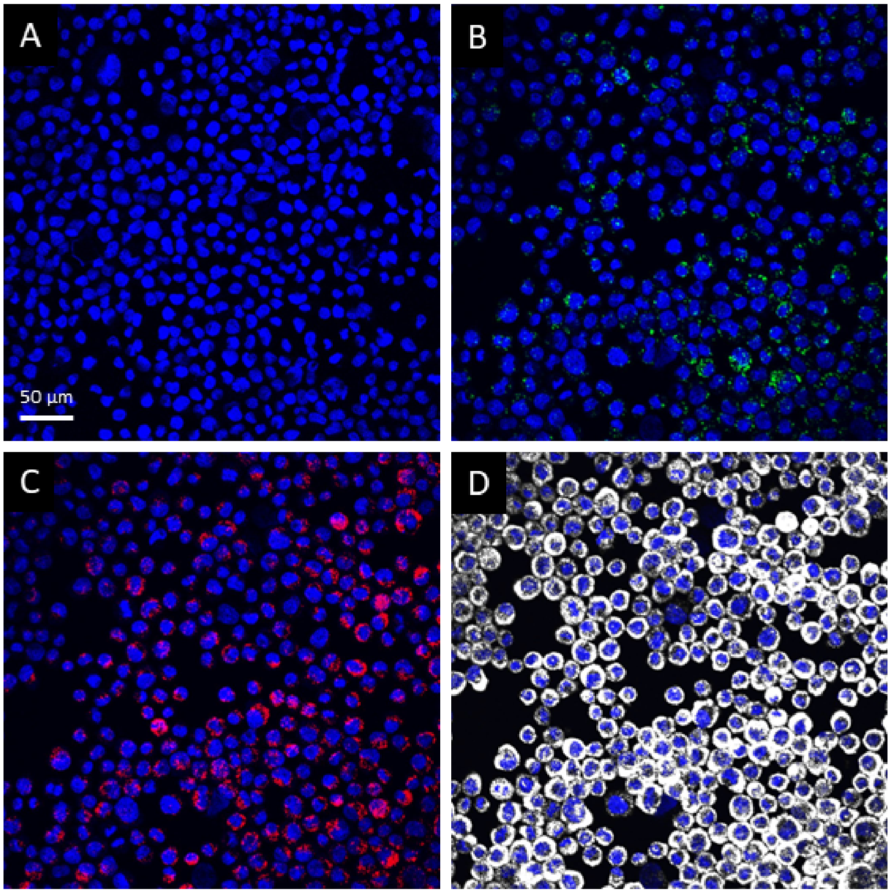


**Figure S3.** RNAscope triplex positive and negative controls in the PE/CA cell line. The RNAscope triplex negative control probes would hybridize with the bacterial dabP gene (**A**). RNAscope triplex positive control probes specific to human *POLR2A* (**B)**, *PPIB* (**C**) and *UBC* (**D**) mRNA targets are shown in green, red and white, respectively. Scale bar: 50 µm for all images.
